# Helminthiases; prevalence, transmission, host-parasite interactions, resistance to common synthetic drugs and treatment

**DOI:** 10.1016/j.heliyon.2019.e01161

**Published:** 2019-01-31

**Authors:** Oladayo Amed Idris, Olubunmi Abosede Wintola, Anthony Jide Afolayan

**Affiliations:** Medicinal Plants and Economic Development (MPED) Research Centre, Department of Botany, University of Fort Hare, Alice, 5700, South Africa

**Keywords:** Pharmaceutical science, Plant biology, Veterinary science, Food science

## Abstract

The morbidity caused by parasite worms on susceptible hosts is of great concern and studies were carried out to explain the mechanism of infection, prevalence, host-parasite interaction and resistance of the parasite to treatment. This review elucidates the prevalence of parasitic worm infections; which is on the increases with the increase in the world population, global warming, poor standard of living particularly in troubled regions and developing nations. The neglect of the disease coupled with the resistance of these parasites to the few available drugs becomes a huge challenge that influences global disease burden. Helminths infections pose a life threat and increase the disability-adjusted life year (DALYs) of the poor and vulnerable people. On the other hand, exploration of medicinal plants as an alternative source of treatment against drugs resistance helminths, attract insufficient attention. This review focused on providing a general overview of the prevalence of helminths, host-parasite interactions, the resistance of helminths and the medicinal plants used to treat helminthic infections.

## 1 Introduction

### 1.1 The parasitic worms

Helminthiases are parasitic worm infections that cause morbidity to their host. They infect man and animals, causing stunted growth and a substantial threat to health. Helminths infection is a huge challenge, both in developing and developed countries due to their continuous contamination of the environment with their eggs and larvae (Nalule et al., 2013). Helminths diseases are one of the most neglected among the healthcare systems. Its neglect could be as a result of it chronic and asymptomatic nature of infection, particularly at an early stage. Helminth infections can, however, cause severe debilitation, morbidity and economic loses among human and livestock. The most common helminths are the soil-transmitted helminths (STH) or intestinal nematodes, filarial worms, schistosomes and onchocerciasis worm. The life cycle of a parasite worm could be very complex, with multiple hosts for different stages; moreover, a major adaptive uttermost parasitism of a worm is a complex life cycle involving trophic transmission (Kasl et al., 2018). The developmental life-cycle of some helminths (soil-borne nematodes) such as Strongyloides and Hookworms have a free-living stage (rhabditiform larvae) and a parasitic stage (filariform larva) which may require a different host or environment.

### 1.2 Symptom and diagnosis

The symptoms of helminths depend on some factors which should be considered during diagnosis. The factors include; the type of worm infection, the duration of infection, the area of infection and the worm burden in the host. The common symptoms of helminthiases are; abdominal pain, diarrhoea, malnutrition, fatigue, enlarged liver and spleen, gastrointestinal inflammation, pneumonitis, blindness, eosinophilia, bowel obstruction, anaemia, vomiting, constipation, lymphedema, weight loss, itchy skin and or anus (Manke et al., 2015). Long-term exposure to helminths and the worm burden in the infected person is directly related to morbidity and severity of the disease, in almost all kinds of worm infections. There are ranges of diagnostic tools for helminthic infection, which include; (1) *Fecal egg examination*: the parasite (s) is identified by microscopically examining the eggs in the faeces of the host, for instance in the case of *Haemonchus contortus, Ancylostoma duodenale, Hymenolepis diminuta* and schistosomes infection (2) *Antigen test*: parasites produce enzymes, hormones and waste that activate the immune response of the host. This response is a biomarker that could be quantified and qualify but this area of diagnosis is still underdeveloped (3) *Serological assay*: the serum of the host is examined for the parasite-specific antibodies, Echinococcosis can be diagnosed using this technique (4) *Nucleic Acid-based Diagnosis*: every organism carries unique DNA sequences. This diagnostic probe is sensitive and specific. It is developed to identify and isolate DNA sequences of different species of parasite. (5) Urine examination: uses point-of-care circulating-cathodic-antigen (POC-CCA) urine test or microscopically examining parasite eggs in host urine, commonly used as a diagnostic tool for Schistosomiasis. (6) *other tools*: could involve physical examination of infected areas, confirmation of parasite hydatid cysts in the tissue of the host in the case of Echinococcosis (Ambrosio and De Waal, 1990; Gougoulias et al., 2010; Manke et al., 2015; Oliveira et al., 2018).

### 1.3 Prevalence of helminthiases

The prevalence of helminth is high in tropical and subtropical areas of the world (Weaver et al., 2010; Tinsley et al., 2011; WHO, 2017) and it is more likely to increase with global climate change especially in the area of poor social infrastructures and lack of sanitation. Hotez et al. (2006), reported an estimate of 187 million people suffers from Schistosomiasis in Sub-Saharan Africa, India, China, East Asia and the Americas. Taylor et al. (2010), reported 90% of the estimated 120 million cases of lymphatic filariasis was caused by *Wuchereria bancrofti* in about 83 countries in Africa, Asia, South and Central America. In 2010, it was estimated globally; 819.0 million (95% Credible Interval (CI), 771.7–891.6 million) were infected with *A. lumbricoides*, 464.6 million (95% CI, 429.6–508.0 million) are infected with *T. trichiura*, and 438.9 million people (95% CI, 406.3–480.2 million) cases of hookworm was reported (Pullan et al., 2014).

It was also documented that helminths undergo adaptation and evolutionary changes due to climatic change or resistance to anthelmintic drugs (Prichard, 2008; James et al., 2009; Nalule et al., 2013; Bauri et al., 2015; Hotez et al., 2016). The adaptation of helminths to this hash environment, encourage the high prevalence of the parasites in the pandemic regions.

### 1.4 Common drugs and resistance of helminths

Helminths are categorized into three major groups: nematodes (roundworms), trematodes (flukeworms), and cestodes (tapeworms). Almost all these parasites could be treated and the level of infection could be reduced below clinical significance with one or a combination of the following categories of anthelmintic drugs; benzimidazoles, macrocyclic lactones, levamisole, piperazine and amino-acetonitrile derivatives (Prichard, 2008; James et al., 2009; Aremu et al., 2012). However, the resistance of helminths to these drugs has been recorded in some literature which is common in the field of veterinary medicine (Prichard, 2008).

The resistance of helminths to drugs poses health complications to both man and animals worldwide. The knowledge of the genetics and mechanisms of helminths resistance to drugs is essential to prevent resistance; to newly developed anthelmintic drugs, to reduce the spread of resistant parasites and to better manage parasite control at all stages of their lifecycle (Prichard, 2008). The resistance of gastrointestinal nematodes and also in others parasites worms such as liver fluke was documented to be high in ruminant (Kaplan, 2004; Wolstenholme et al., 2004; Prichard, 2008; Morgan et al., 2013). It is, therefore, necessary for parasitological exploration of the mechanisms of anthelmintic resistance in other to develop alternative treatment approaches and drugs for the control of helminths. The main strategies leading to the discovery of new anthelmintic drugs were mainly based on the screening of new drugs via an *in vitro* and *in vivo* test systems (Prichard, 2008; James et al., 2009).

### 1.5 Alternative cure and drug resistance

Resistance to each of the categories of anthelmintic drugs has been reported and there is a need for new drugs with different mechanisms of action (James et al., 2009). Plants produce a broad spectrum of secondary metabolites or phytochemicals which aid in several biological activities including the defence of the plant against pests and diseases. The major classes of phytochemicals include phenolic, alkaloids and terpenoids compound. These phytochemicals make some plants a good source of remedy for ailments. Plant secondary metabolites have been successfully used in ethnomedicine and are generally used for; insecticide, piscicidal, molluscicidal, antimicrobial, antiparasite and other ailments. The global demand for herbal medicines is rapidly on the increase (Kuria et al., 2012). The out-of-pocket spending on complementary health services and herbal products was estimated to US$83.1 billion in 2012 and US$14.8 billion in 2008, in China and the United States of America respectively (Qi Z. and Kelley, 2014).

A relative number of medicinal plants have been reported to possess anthelmintic activity in modern medicine and also utilized by folk ethnic groups worldwide. Several medicinal plants, have been identified following the folk medicine claims and the isolated phytochemicals have been scrutinized for their anthelmintic activities in the search for novel anthelmintic drugs (Yellasubbaiah et al., 2015). There are several promising results obtained from *in vitro* and *in vivo* studies of antihelmintic medicinal plants but few or none of these results was translated into clinical practice (Wink, 2012). In this review, focus is on parasitic worms, its prevalence, resistance to drugs and anthelminthic medicinal plants previously highlighted by various researchers, in the attempts to evaluate its efficacy for treatment of helminths.

## 2 Main text

### 2.1 Methodology of the review

#### 2.1.1 Data source and search strategy

In this review, literature was search electronically using the information from search engines. The articles were searched on databases; Web of Science (WOS) and Scopus using the queries “Parasitic worms OR anthelminthic plants OR helminths”. The search was from inception to December 31, 2017, for studies describing and covering the following areas; ethnomedicine, ethnobotany, ethnopharmacology, ethnogeography, phytochemistry, pharmacology, genetics and parasitology of helminths. The literature search was however not geographical or regional based. Furthermore, the references of the resultant research work and review articles were screened to identify the type of studies. Duplicated literature was excluded and only complete study was considered for this review.

#### 2.1.2 Inclusion criteria

Agricultural, biological, environmental, Plant science, genetics and molecular biology studies, related to helminths were included. Both interventional and observational studies were considered within the scope of this review. Articles of anti-nutrient and resistance of helminths were also considered relevant for this review.

#### 2.1.3 Exclusion criteria

Articles written in other languages aside from English, as well as animal studies, were excluded. Book series, proceeding papers, case reports and studies containing less and irrelevant subjects were also excluded. Articles addressing the prevalence of helminths although relevance but was regional based were considered outside the scope of this review.

#### 2.1.4 Result

**Fig. 1:**
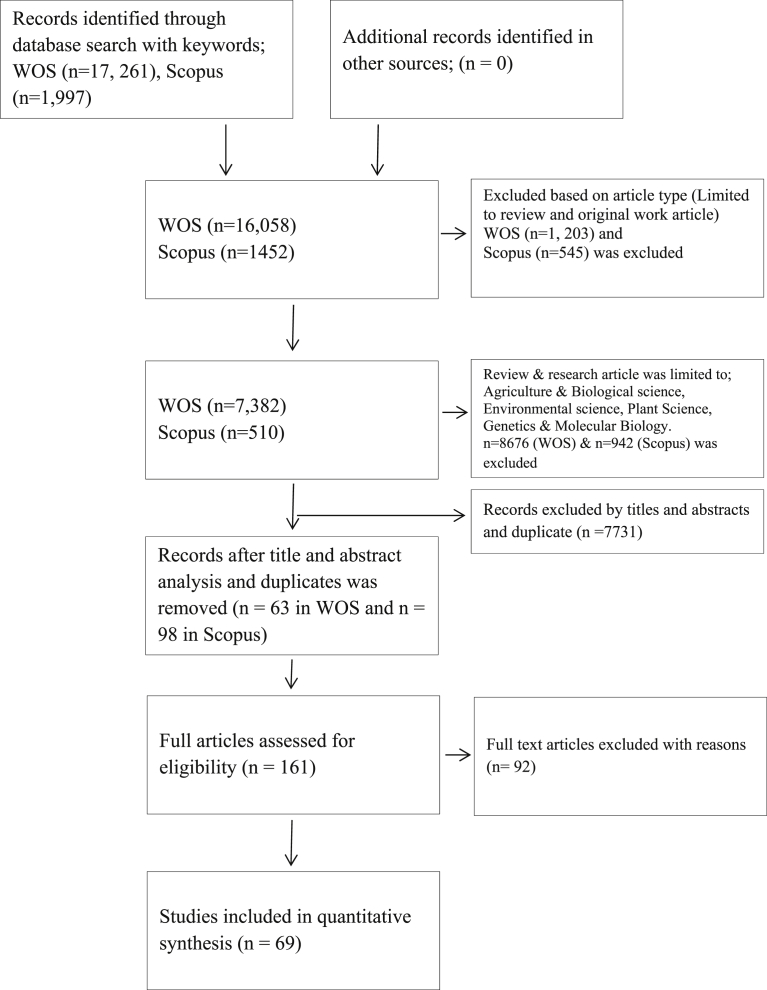
Flowchart showing the literature search, article screening, elimination and final inclusion. n: number of articles.

*Literature search*: A total of 19,258 articles were retrieved initially, in the database of Scopus and Web of Science. The articles were then limited to reviews and research article, in which a total of 1,748 articles was excluded. The literature was then limited to Agriculture and biological science, Environmental science, Plant Science, Genetics and Molecular Biology, resulting in the elimination of 9,618 articles. A total of 7,731 articles were excluded based on the title, abstract and duplicates. After reviewing the full-text, 92 articles were excluded. Finally, 69 studies were considered eligible for this review (Fig. 1).

### 2.2 Distribution of helminths

The level of infection and prevalence of intestinal worm is an indicator of the climatic and living conditions in a society (Kumar et al., 2014). Khanum et al. (2010), reported the relationship between intestinal helminths burden and the variation in climatic conditions among outdoor patients. They recorded the lowest prevalence (19.38%) in the months of winter and highest (29.3%) during the rainy season in Bangladesh. Similar research was carried out in India by Kumar et al. (2014), supporting the burden of intestinal parasites according to Khanum et al. (2010). Kumar et al. (2014), reported the highest prevalence (80.5%) in autumn and lowest (43.9%) in the months of spring.

Satellite data using Geographical information systems (GIS) and Remote sensing (RS) of Land Surface Temperature (LST) has been used to reveal the large-scale distributions of few helminths infection, which was confirmed to be influenced by climate (Brooker et al., 2006; Hotez et al., 2008). For instance, It was reported, using satellite data, the prevalence of hookworm infection remains highly prevalent in areas where LST exceeds thermal range 38–40 °C whereas the prevalence of *Ascaris lumbricoides* and *Trichuris trichiura* was reduced averagely by 5% in such area (Brooker et al., 2006). This explains how enormously weather and temperature could contribute to the prevalence of helminthic infections. Therefore, the prevalence and distribution of parasitic worms infections in tropical and subtropical areas, with the greatest numbers occurring in sub-Saharan Africa, the Americas, China and East Asia as shown in Table 1, could be as a result of the suitable weather conditions (Hotez et al., 2008).

**Table 1:** Global prevalence of major human helminths and regions.

Disease	Major parasite	Global prevalence (Million)	Regions of prevalence	Reference
**Soil-transmitted Helminths**				
Hookworm	*Necator americanus*; *Ancylostoma duodenale*	740–1300	Latin America, Sub-Saharan Africa, South Asia, and the Caribbean	Kumar et al. (2014); WHO, 2017
Ascariasis	*Ascaris lumbricoides*	1221–1472	Southeast Asia, Sub-Saharan Africa, and Latin America	Kumar et al. (2014); WHO, 2017
Strongyloidiasis	*Strongyloides stercoralis*	30–100	Tropical and subtropical countries in Asia, Africa, and Latin America	Hotez et al. (2008)
Trichuriasis	*Trichuris trichiura*	750–1050	Tropical Asia, Africa and South America	Kumar et al. (2014); WHO, 2017
**Filarial nematodes**				
Lymphatic filariasis	*Brugia malayi; Wuchereria bancrofti*	120	Tropical and subtropical regions of Africa, Asia, South and Central America and Nations of Pacific Island	Taylor et al. (2010)
Loiasis	*Loa loa*	13	West and Central Africa	Hotez et al. (2008)
Onchocerciasis (river blindness)	*Onchocerca volvulus*	37	Sub-Saharan Africa, Yemen and isolated areas of South America	Hotez et al. (2008)
Dracunculiasis	*Dracunculus medinensis*	0.01	Chad, Ethiopia and Mali	Hotez et al. (2008)
**Platyhelminth flukes**				
Schistosomiasis	*Schistosoma mansoni; Schistosoma haematobium*; *Schistosoma japonicum*	200–209	Africa, Middle East, Brazil, Venezuela, the Caribbean, Suriname, China, Indonesia, the Philippines and France	WHO, 2012
Food-borne trematodiases	*Clonorchis sinensis*; *Fasciolopsis buski*; *Opisthorchis viverinni; Paragonimus spp*; *Fasciola hepatica*	56	East Asia and South America	Torgerson et al. (2014)
**Platyhelminth tapeworms**				
Cysticercosis	*Taenia solium; Echinococcus species; Taenia saginata*	0.4	Latin America, West Africa, India, Russia, North-East China, Pakistan and Southeast Asia	Hotez et al. (2008); Torgerson et al. (2014)

#### 2.2.1 Factors influencing the distribution of helminths

Factors influencing the patterns of dispersion in the distribution of parasitic worms within host populations are numerous. The demographic process of parasite populations in related to distribution was argued to be dynamic. Hence, transmission of parasite to the next host is essential components of parasite fitness to survival, which could be influenced by host/parasite genome, host defences, parasite virulence, environment and prevalence of parasite (Ulrich and Schmid-Hempel, 2015). Human, animals and fish are the major hosts of helminths, and infection could be transmitted interspecies or intraspecies. It is therefore imperative to understand the richness of helminths and the factors that influence it, in other to understand the prevalence and its distribution. Kumar et al. (2014), recommended that the prevalence of helminths infection in a population or sample of the same species can be calculated by the formula;

$$\text{Prevalence}=\frac{\text{Number}\;\text{of}\;\text{subjects}\;\text{testing}\;\text{positive}\;}{\text{Number}\;\text{of}\;\text{subjects}\;\text{investigated}}\;\times \;\frac{100}{1}$$

The biogeographical and sustainability of parasite are highly influenced by host specificity. That is; host habitat, niche and behavioural survival are part of factors that determined the success of parasite to invade a new host or its adaptation to the previous host. Therefore, the interaction between host, parasite and their environments determined the prevalence of the regional helminths fauna (Poulin and Mouillot, 2003; Salgado-Maldonado et al., 2016). For instance, Salgado-Maldonado et al. (2016), determined the distribution of helminths in a community of freshwater fishes in Neotropical river Mexico, using a randomized sampling of species accumulation curves to determine the asymptotic richness based on Clench's model equation;

$${\text{V}}^{2}=\frac{\left(\text{a}\times \text{n}\right)}{(1+(\text{b}\times \;\text{n})\;}$$

The gradient of the cumulative species curve was determined by;

$$\text{s}\;=\frac{\text{a}\;}{{\left(1+\text{b}\times \text{N}\right)}^{2}\;}$$

where; n = number of hosts examined in a species, V^2^ = observed richness, a = rate of adding species, b = parameter related to the shape of the curve, s = slope and N = total number of hosts examined.

#### 2.2.2 Mode of transmission of helminths

Parasitic worms could be transmitted to their host through these major ways; (i) *Direct method*: Transmission is by host direct contact with eggs without intermediaries. The eggs of the helminths are passed through feces, it hatches into larvae and re-infect its host or other hosts, example; *Enterobius vermicularis* (Arkoulis et al., 2012). (ii) *Modified direct*: The eggs of the helminths are passed in feces of the host during open defecation to the soil and undergoes developmental stages, after some days. The egg could be ingested by a susceptible host directly from the soil or in food, afterwards hatch into larvae and enter blood circulation by penetrating the stomach wall. When it reaches the lungs, it is coughed out and re-swallowed back to the intestine where it grows into an adult. *A. lumbricoides* and *T. trichiura* are examples of worms in this category (Jourdan et al., 2018). (iii) *Skin Penetration*: The eggs of these adult helminths are shedded and passed in feces or urine of the host into the soil or fresh water. It either develops into infective larvae freely or with the aid of an intermediary host, depends on the type of parasite. It then penetrates the skin of a susceptible host and gets into the blood circulation. For instance, the larvae of *A. duodenale* and *S. stercoralis* find their way to the lungs, re-swallowed by the host and becomes an adult in the intestine (Jourdan et al., 2018). On the other hand, the larvae of worms that causes Schistosomiasis in humans remains in the blood vessels until it developed into adult phase, the adult worm resides in the mesenteric venules in various locations of the body (Giannelli et al., 2016). (iv) *Bite (s) from infected insect vectors*: carrier agents such as mosquitoes in Lymphatic filariasis, deer fly in Loiasis and black fly in the case of Onchocerciasis, transmit the parasitic worms by biting and deposit infectious larvae of the parasite in or around the wound. The larvae mature into adults under the skin (subcutaneous tissue), where they produce microfilaria that migrates to the skin waiting to be taken during blood meals by another vector and then developed into infective larvae in the gut of the insect to restart the cycle. (v) *Foodborne helminths*: host becomes infected by consuming poorly cooked or uncooked fish, vegetables, crustaceans, meat or drinking water that is contaminated with the parasite larvae, eggs or cysts. Cysticercosis, Dracunculiasis, Ascariasis and Food-borne trematodiases are examples of helminthic diseases that can be contacted from food (Torgerson et al., 2014).

#### 2.2.3 Host-parasite interactions

**Fig. 2:**
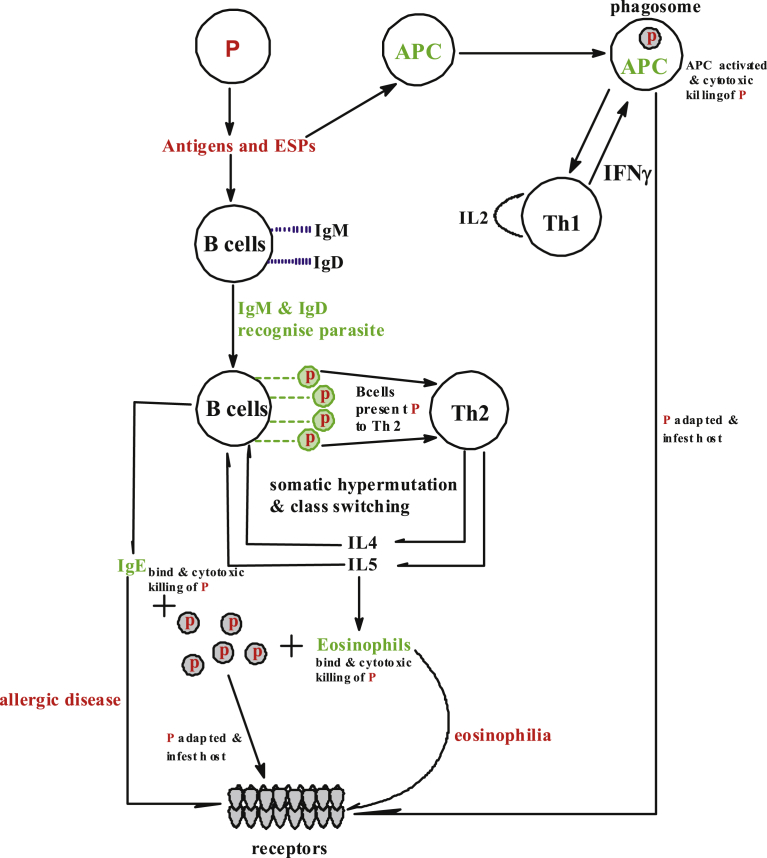
Host-parasite interaction; helminths mechanism of survival is by the production of antigens, ESPs and blockage of IL-33R of the host by interacting with alarmins receptors ST2. The innate mechanism of the host response to helminths invasion, firstly by code recognizing parasite using antibodies IgM and IgD from B cells. Then Th2 initiate and stimulate eosinophils and IgE that bind to the helminths causing cytotoxic. The antigens of the parasites are also recognized by antigen presenting cells (APC) which bring about the autonomous response of Th1. In most cases, the parasite survives in the host by immunomodulating the host's immune system. P: helminths parasite, APC: antigen presenting cells, IL: interleukin, IFNγ: Interferon gamma, Ig: immunoglobulin, Th: T-helper.

Host-parasite relationship has been existing for ages; parasites are beneficiary in this relationship. They ensure survival within or around the host by molecular adaptations that down-regulate immune responses of the host and manipulate host gene expression. Helminths especially, take charge of the host immune system by firstly mimic the immune antigen of the host, deactivate the host detection systems that would otherwise trigger the autonomous immune response and then subdue the immune system (Maizels et al., 2012; Coakley et al., 2016), hence it becomes difficult for the host to auto-eliminate the parasite (Fig. 2). Whenever parasitic worms such as platyhelminths (flatworms) and nematodes (roundworms) invade their host, there is a need for hemostasis and outwitting host immune defences and expulsion mechanisms. The helminth produces excretory-secretory (ES) antigens which mediate a protein-protein interaction in the extracellular ambience with either the fluid phase or host receptor cells. For instance, *Heligmosomoides polygyrus* secretes cytokine TGF-β in this regards as a means to win the host immune system to itself (Coakley et al., 2016). ES production is not to overtake the immune system but to modulate and manipulate the immunity of the host to their own favour.

The autonomous immune system of the host, on the other hand, respond to the invasion of the parasite by inducing the production and mobilization of certain proteins such as; cytokines (interleukin-13, interleukin-10, interleukin-5, and interleukin-4), non-specific immunoglobulin E, parasite-specific immunoglobulin, mobilization of mast cells, basophils and eosinophils. These collections of responses to helminths are known as the T-helper-2 (Th2) immune response (Maizels et al., 1993; Bethony et al., 2006; Hotez et al., 2008). The Th2 immune response involves the immediate activation of adaptive (CD4^+^ T cells) and the innate (basophils and eosinophils) cells (Hotez et al., 2008). The immune of the host can also recognize helminthic invasion with the aid of antigen presenting cells (APC), which is Th1 specific signal protein. Th1 self-initiates IL2, which enable the antibodies to produce Interferon gamma (IFNγ) cytokines that fight the helminths at an early stage. The host effector's response to Th2 and Th1 can bring about some physiological changes in the organism such as increased mucus secretion, cell hyperplasia, muscle contraction, allergy and eosinophil-mediated tissue inflammation (Fig. 2) (Bethony et al., 2006). The host responses to helminths invasion have made it possible to qualify and quantify parasite infections in the serum by the use of these responses as a biomarker. However, the area of bio-marking still requires development.

#### 2.2.4 Excretory-secretory products of helminths

Parasitic worms in the tissue or gastrointestinal tract of a host usually respond to the host immune system by simply blocking immune responses of the host via secretion of some hormones and waste called Helminth Excretory Secretory products (HESP). Research reveals that the homeostatic response of the helminths to the host immune could cause suppression of host lymphocytes which ensure the survival of the helminth, as reported in the case of *W. bancrofti* (Harnett, 2014).

However, as research goes further into HESP, it was discovered that the effects of helminths on the host's immune once documented is “immunomodulation” rather than the “immunosuppression” reported (Harnett, 2014; Smallwood et al., 2017). This brought about a new interest in the research on how helminths could be used for therapy rather than outright condemnation. Immunomodulation caused by helminths is currently recognized as an occurrence common across the host of numerous parasitic worms. Immunomodulation causes two major features in the host (i) stimulate cytokines to form signal proteins such as regulatory T-(Treg), B-(Breg) cells, transforming growth factors (TGF) and anti-inflammatory IL-10 (ii) It induces the mast cells, T helper 2 (Th2)-immune response including cytokines IL-4, IL-5 and IL-13, antibody Immunoglobulin E (IgE), secretion of eosinophils and mastocytosis. All or few of these modulations may act to suppress the antigens secreted by the parasites but eventually resulted to protective responses which prevent possibly potential dangerous pathogens and parasites from invading the host (Harnett, 2014; Sawant et al., 2014; Smallwood et al., 2017). This has attracted interest among immunologists, as this could lead to a novel therapy for helminthiases, other diseases and the development of an antiparasite vaccine.

### 2.3 Synthetic anthelminthic drugs and resistance

Pathogenic bacteria, parasites or cancer with initial successful drug treatment may rapidly be tolerant or resist drug due to mutation or other physiological changes. Hewitson et al. (2009), argued that resistance could be said to have occurred when a susceptible population shows any decrease in response to treatment at the expense of tolerable dose of the drugs by the host. Resistance is becoming an emerging problem in many filed of medical science. It is, therefore, necessary to comprehend the mechanisms by which resistance is developed in these diseases so that alternative therapeutic strategies could be developed.

Drugs are the current tools to control worm infections. It was documented between the year 1975–2004, 1556 chemicals drugs have been marketed to treat helminthiases (Hotez et al., 2008). The drugs are of broad spectrum which is used for both human and animal or either and are divided into classes based on their mode of action and chemical structure; (1) benzimidazoles (BZs)[(oxfendazole, fenbendazole, albendazole, abamectin and mebendazole etc] (2) macrocyclic lactones (MLs) [moxidectin, eprinomectin, ivermectin, and doramectin etc.] (3) levamisole (LEV) [pyrantel and morantel] (4) amino-acetonitrile derivatives (AADs) (Prichard, 2008; James et al., 2009; Aremu et al., 2012). (5) Piperazine (PZ) [Diethylcarbamazine] (6) Paraherquamide [Marcfortine A and Paraherquamide A].

#### 2.3.1 Drugs mode of action and parasite resistance

The mode of action of most anthelmintic drugs depends on the affinity of the parasite's signal protein, anion and cation channels and neuromuscular receptors to the drug. The drug binds to these sites and causes instability, spastic paralysis, or death of the parasite. On the other hand, there have been reported cases of resistance of certain strains of the parasites to anthelminthic drugs (Prichard, 2008; James et al., 2009). The resistance of helminths to drugs is a challenge that requires the development of novel drugs that uses a different mode of action compared to the previous anthelminthic drugs.

##### 2.3.1.1 Benzimidazoles

Benzimidazoles were introduced into the commercial market in 1961 and resistance was reported in 1964 (James et al., 2009). These drugs bind to the ß-tubulin dimers of the growing end of microtubules of the parasite, preventing microtubules from polymerization and eventually caused the death of the parasite. The success of benzimidazoles in *Caenorhabditis elegans* was studied to be mediated by a single gene, *ben-1*, which is encoded in ß-tubulin (Prichard, 2008; Aremu et al., 2012).

##### 2.3.1.2 Levamisole

Imidazothiazole drugs (levamisole) were released to the market in 1970 but the first resistance was reported in 1979 (James et al., 2009). LEV and related compounds target the nicotinic acetylcholine receptors (nAChR), which act as an agonist's compound at the neuromuscular receptor, resulting in spastic paralysis and egg-laying as recorded in *C. elegans*. In this regards, intestinal parasitic worms could be killed by the drug or pushed out by peristalsis movement of the intestine.

##### 2.3.1.3 Macrocyclic lactones

MLs (ivermectin) was released in 1981 but by 1988 there are cases of resistance (James et al., 2009). MLs interfere with the gamma-aminobutyric acid (GABA)-mediated neurotransmission at the glutamate-gated chloride channels (GluCl) of the helminths, including α7 nACh receptors (Wolstenholme and Rogers, 2005). Moreso, nematode glutamate-gated chloride channels (GluCl) has a high affinity for ivermectin, this correlates with the anthelmintic potency of the drug (Holden-Dye and Walker, 2007).

##### 2.3.1.4 Amino-acetonitrile derivatives

AADs are novel synthetic anthelmintics drugs recently discovered and tested experimentally for veterinary use only, at the moment. A newer version of the drug, AAD-1566 (monepantel) was to be developed for better pharmacological actions (Rufener et al., 2009). AADs are the group of compounds with a low molecular mass that is easily accessible by Strecker reaction and acylation of the amine with aroyl chlorides and the alkylation of phenols with chloroacetone. They bear different aroyl moieties and aryloxy on an amino-acetonitrile core (Kaminsky et al., 2008a, Kaminsky et al., 2008b; Rufener et al., 2009; Aremu et al., 2012). The mode of action of AADs involves the ability of the drug to interfere with a unique clade of acr-23 nicotinic acetylcholine receptor subunit which causes paralysis of the worms. At the moment, AADs overcomes existing resistances of worms to the current available anthelmintic drugs and are well tolerated with low toxicity to mammals (Kaminsky et al., 2008a, Kaminsky et al., 2008b; Rufener et al., 2009; Aremu et al., 2012). Recent reported reveals that Monepantel (AAD-1566) is a selective drug which has low or no activities against some relevant STH such as *Ascaris suum*, *Necator americanus*, *Strongyloides ratti*, and *Trichuris muris* (Tritten et al., 2011). AADs are therefore not suitable for all helminthic infections, which is still a challenge in the treatment of helminths infections.

##### 2.3.1.5 Piperazine (PZ)

It was commercialized in the 1950s and it is suitable for the treatment of filariasis and threadworm. Its mode of action; it acts as an antagonist against GABA receptors and causes flaccid, reversible paralysis of helminths body wall muscle as studied in *Ascaris suum* (Holden-Dye and Walker, 2007)*.*

##### 2.3.1.6 Paraherquamide

Marcfortine A and Paraherquamide A are isolated from *Penicillium roqueforti* and *Penicillium paraherquei* respectively. Both drugs belong to members of the oxindole alkaloid family. In studies, Marcfortine A was confirmed to be active against *C. elegans* at a high dose. On the other hand, Paraherquamide and its derivatives were found to induce flaccid paralysis in many parasitic nematodes during *in vitro* studies (Lee et al., 2002).

#### 2.3.2 Genomic coding of helminths and vaccination

Due to improving techniques in RNA and DNA extraction, advances in instrumentation for DNA sequencing and the construction of whole genome libraries and complementary DNA (cDNA); helminths genome sequencing have improved immensely. Genomic coding of helminths began with the analysis of expressed sequence tags (ESTs) or transcribed sequences, which grew rapidly. Brindley et al. (2009), reported about 450, 000 Platyhelminthes and 550, 000 nematodes ESTs was already in the GenBank of dbEST division as at April 2009. ESTs have been so helpful in the field of parasitology. It can be used to interpret helminths genomes; track mutation along filial generations, confirm intron/exon, mark gene boundaries and confirm open reading frames (ORF) (Brindley et al., 2009).

The gene coding of helminths is complex because the parasites have large genomes, unlike unicellular organisms. Whole genome sequencing of a parasite has a size ranges from 50 to 500 Mb, with up to 20,000 protein-encoding genes which could be compared to that of insects (Hotez et al., 2008). Several helminths genome has been analyzed, examples include; *Brugia malayi* (lymphatic filarial worm), *Ancylostoma caninum* (Dog hookworm), *Schistosoma japonicum* (blood flukes) and *Schistosoma mansoni* (Müller-Myhsok et al., 1997; Brindley et al., 2009). The whole genome sequencing of helminth causing schistosomiasis was mapped and it confirmed, there was a linkage of the occurrence of *S. mansoni* infection to the Brazilians in the chromosomal 5q31–q33 region and subsequent affirmation of this link was recognized in a Senegalese population (Müller-Myhsok et al., 1997; Hotez et al., 2008). This is an indication that, the successful mapping of the whole genome of parasitic helminths could promote new therapeutic approaches or drugs for the treatment of the disease, including other diseases linked to helminth pathogens.

The resistance of helminths to drugs over the years generated a hypothesis on whether preventive chemotherapy could be used against helminthiasis. This led to the development of vaccine antigens for several helminths. The vaccine for trichuriasis and ascariasis are currently undergoing preclinical testing. Hookworm, schistosomiasis, onchocerciasis and other STH infections vaccines are in various stages of development (Hotez et al., 2016). There have been reports that helminths can influence the efficacy of vaccines even when it is targeted against other diseases or parasite, by modulating host immune system especially when cellular-dependent and Th1 responses are required. For example, the infection of *Onchocerca volvulus* and Schistosoma species was reported to decrease the effect of vaccine used to treat tetanus and tuberculosis (Moreau and Chauvin, 2010). In a rat model, it was confirmed that the immunity of rat against *Plasmodium chabaudi* could be turned down by *Heligmosomoides polygyrus* infection (Su et al., 2006). Therefore, in the development of new vaccines, the immunomodulatory capacity of parasitic worms must be taking into consideration in other to counterbalance resistance and increase the efficacy of the vaccine.

#### 2.3.3 Mechanism involve in helminths resistance

##### 2.3.3.1 Single-nucleotide polymorphisms (SNPs)

Drugs resistance in helminths has been identified to alteration of the drug's cellular target, by changes in the genome sequence. Several cases of SNPs have been linked to resistance in helminths. SNPs are unique genetic differences between individual organisms in a population which usually arises by gene mutation after the treatment of drug. Such a change can lead to an amino acid deletion or substitution in the drug target protein which hinders the affinity of the drug to the binding site (James et al., 2009). This single nucleotide change could spread or transfer to the next filial generation which brings about strains of helminths which are tolerant or resistant to a particular drug. The most common resistance in BZs caused by SNP as studied in *Haemonchus contortus* is the change in the ß-tubulin binding site (TTC to TAC) which changes at codon 167 to 200 (phenylalanine to tyrosine) (Prichard, 2001).

##### 2.3.3.2 Multidrug resistance (MDR)

P-glycoprotein (P-gp) is found in the epithelial cells and has excretory roles. It aids multidrug resistance (MDR) by active transport of drugs using the energy derived during the binding or hydrolysis of ATP. P-glycoprotein belongs to ATP-binding cassette (ABC) transporter subunit (ABCB1), several ABC transport proteins are known to aid MDR of different drugs (James et al., 2009). It is able to transport a wide variety of lipophilic substances, including a different spectrum of drugs. It decreases the amount of drug reaching its target site and consequently reduces the effect of the drug hence resulting in resistance (James et al., 2009). This mechanism of resistance was confirmed in the study of ivermectin and levamisole against *Haemonchus contortus* by Molento and Prichard (1999).

##### 2.3.3.3 Antioxidant enzymes

Many drugs used for the treatment of parasitic worm infections releases free radicals as part of the mechanism of cytotoxic. A retort release of antioxidants such as glutathione and other enzymes decreases the cytotoxic effect of the drug on parasite and hence resistance. It was speculated that premature schistosomes are more susceptible to drugs than the adult because they have lesser free radical scavenging enzymes (Hewitson et al., 2009; James et al., 2009). It was confirmed after the administration of Cambendazole to adult *Haemonchus contortus* that the level of Glutathione S-transferase was increased in the parasite for scavenging of free radicals released by the drug and thereafter aid resistance of the helminth to the drug (Kawalek et al., 1984).

### 2.4 Anthelminthic medicinal plants

Several medicinal plants have been claimed by folklore medicine and confirmed scientifically to possess anthelmintic activities. Plants with such medicinal values are used worldwide by various ethnic groups for similar or same purposes (Yellasubbaiah et al., 2015). Several medicinal plants with anthelminthic potentials have been documented and such plants could be in the subcategories; epiphytes, spices, herbs, vascular plants, vegetables and shrubs (Yellasubbaiah et al., 2015; Jimoh et al., 2018). The confirmation of the anthelmintic properties of most plants is primarily from ethnoveterinary sources, as the efficacy is been tested using animals *in vivo* studies. Ethnoveterinary medicine (EVM) provided concrete evidence and information on plant preparations, usage and documentation in different parts of the world (Table 2) (Githiori et al., 2006). The anthelmintic properties of medicinal plants can be validated by the exploration of such plant remedies using both *in vivo* and *in vitro* studies. There have been reports that *In vitro* assays are a useful tool for a pre-screens evaluation of bio-activity of anthelminthic plants but concentrations of plant extract used during *in vitro* studies do not always correspond to *in vivo* bioavailability (Githiori et al., 2006).

**Table 2:** Anthelmintic Medicinal plants; it active principles and anti-nutrients.

Plant	Anthelmintic active principle (s)	Part used	Tested organism	Anti-nutrient	Reference (s)
*Carica papaya* L.	papain (papaya proteinase I) and Benzyl isothiocyanate	Leaves, fruit and seed	*Trichostrongylus colubriformis, Ascaris suum*	phytate, oxalate, condensed tannin and hydrolysable tannin	Hounzangbe-Adote et al. (2005); Adetuyi et al. (2008); Bauri et al. (2015)
*Butea monosperma* Lam*.*	Palasonin and Tannins	Seed, root, flower and leaves	*Ascaris lumbricoides, Dipylidium caninum, Toxocara canis*	Protease inhibitors	Mishra et al., 2000; Pandey and Jamal (2010); Bauri et al. (2015)
*Terminalia arjuna* Wight and Arn.	Tannin and ellagic acid	leaf, bud, seed, root and stem	Eggs, larvae and adult of *Haemonchus contortus*	Phytic acid, tannin	Bauri et al. (2015)
*Allium sativum* L.	Allicin	bulbs,	*Ascaridia galli, Haemonchus contortus*	Hydrocyanic acid, oxalate, phytic acid, cyanogenic glycoside	Kavindra Singh and Nagaich, 2000; Iqbal et al. (2001); Asanga et al. (2015)
*Cucurbita maxima* Duchesne	Cucurbitin	Seeds	*Ascaris lumbricoides, Hymenolepis diminuta*	Akaloids, saponins, Tannin, Cyanide, Oxalate, phytate	Iqbal et al. (2001); Mohaammed et al. (2014)
*Zingiber officinale* Roscoe	Zingiberene, bisabolene, gingerols and shogaols	Rhizome	*Haemonchus contortus*	Tannin, Phytin and Oxalate	Iqbal et al. (2001); Adanlawo and Dairo (2007)
*Nigella sativa* L.	Thimoquinone and Dithimoquinone	Seeds	*Schistosoma mansoni*	Trypsin inhibitor, phytic acid	Kailani et al. (1995); Bauri et al., 2015
*Piper longum* L.	piperine	Fruit and leaves	*Ascaris lumbricoides*	Tannin	Bauri et al. (2015); Singh et al. (2015)
*Ocimum sanctum* L.	Eugenol, ß-caryophyllene and Urosilic acid	Leaf	*Caenorhabditis elegans, Ascaridia galli*	Phytin, Oxalate, Tannic acid, Phytic acid	Asha et al. (2001)
*Azadirachta indica* A. Juss.	Azadirachtin	Leaves	*Fasciola gigantica*	condensed tannins, crude saponins, oxalate, lignin, azadirachtin	Kushwaha et al. (2004); Adjorlolo et al. (2016)
*Moringa oleifera* Lam.	Tannins, Flavonoids, Triterpeniods, Saponins and Alkaloids	Leaf and seed	*Dracunculus medinensis*, *Schistosoma mansoni*	Oxalates, Alkaloids, Phytate, Tannin	Stevens et al. (2015)
*Artemisia annua* L.	*Artemisinin, Quercetin*	Leaf	*Fasiola hepatica, Schistosoma mansoni*	Phytate, Tannin	Brisibe et al. (2009); Dragan et al. (2014)
*Cymbogon martini* (Roxb.) Wats.	Geranio	Whole Plant	*Caenorhabditis elegans*	–	McGaw et al. (2001)
*Mentha cordifolia* Lej. & Courtois auct.	Glucosides, b-Sitosterols,	Leaf	*Ascaris suum*	–	Villaseñor et al. (2002)
*Calotropis procera* (Aiton) W.T. Aiton	Anthocyanins, Triterpenoids, alkaloids	Leaf	*Haemonchus contortus*	Phytic acid, Tannins, Oxalate	Adebayo et al., 2015, Al-Qarawi et al., 2001
*Aloe ferox* Mill.	Phenol Saponin Alkaloids Flavonoids	Leaf	Trichuris spp.	Tannin, Oxalate, Phytate	Adesuyi and Awosanya (2012); Maphosa and Masika (2012)

#### 2.4.1 Safety and a possible side effect of anthelminthic plants

There is concern about the scientific evidence through *in vitro* and *in vivo* test of antihelminthic plants efficacy, pharmacokinetics and safety, irrespective of their wide usage. The possible side-effects of plant products are not ruled out prior to their adoption as a novel source of parasite control (Githiori et al., 2006). The phytochemicals responsible for the biological activities of the medicinal plant is produced in broadband of secondary metabolites. The major classes of the secondary metabolites are terpenoids, alkaloids, phenolic compounds, non-protein amino acids and other polyphenols are considered responsible for the anti-parasitic effect of plants and probably responsible for the possible side effect (Akinsanya et al., 2016; Idris et al., 2017).

Irrespective of the wide usage and acceptance of medicinal plants, the scientific evidence on the efficacy and side effect of most anthelminthic plants is limited (Githiori et al., 2006). Scientific evaluation of medicinal plant pharmacokinetics is still at the embryonic stage and hence the dose required for treatment of a particular ailment is difficult to quantify. This is due to the variation of the phytochemicals content of plants within species and species across geographical regions. Environmental differences play an active role in the therapeutic attributes of a plant. The phytochemicals content must, therefore, be ascertained scientifically considering all factors as this is necessary prior to plant products adoption as a novel source for parasite control.

The dose of a synthetic drug and it pharmacokinetics can easily be evaluated because it is a pure compound, unlike plants phytochemicals. There have been reported cases of allergies and side effects in both short and long-term usage of herbal anthelminthic drugs (Ferrara et al., 2011). Some active compounds in plants may cause a negative physiological effect on consumers and may also contribute to antinutritional factor as shown in Table 2. Antinutrients are phytochemical compounds that interfere with nutrients absorption and may also interfere with certain enzymes in the body. Anti-nutrient such as; amylase inhibitors, found in kidney beans, prevent the breakdown of the glycosidic bonds of starches and protease inhibitors substances found in soybeans, prevent pepsin, trypsin, and other protease from digesting protein. Furthermore, there have been controversial reports that phytochemical such as; Capsaisin (Chili Pepper), phytoestrogens genistein (found in; soybeans, red clover, fava beans) and Ptaquiloside (Bracken Fern) have potential carcinogenic effects (Bode and Dong, 2015). These pose quite a huge challenge in the acceptance of herbal remedies which required the effort of identification, isolation, advancement, management and validation of these effects to encourage their wider acceptance. Some plant secondary metabolites such as alkaloids, saponins, non-protein amino acids, tannins, lignins, glycosides and other polyphenols have been considered to be responsible for the anti-parasitic properties of some plants but they are also reported to contribute to the anti-nutrient effect of these plants as shown in Table 2 (Githiori et al., 2006).

#### 2.4.2 Health implications of helminths

Parasitic worms remain an international menace and medical neglected disease, despite their economic and public-health important. They cause one of the world's most devastating diseases which influence the host's body physiologically, physically and psychologically (Bethony et al., 2006). Helminthic infections are more common in developing countries; it poses a large threat to the public health and contributes to the prevalence of anaemia, malnutrition, eosinophilia, pneumonia, diarrhoea or dysentery, and reducing absorption of micronutrients. Aside from the general symptoms, some helminths such as tapeworms can cause health complications such as cysticercosis, neurocysticercosis, and echinococcal disease (Torgerson et al., 2014). Schistosomiasis is caused by the parasitic worms in the genus *Schistosoma,* and they are known for the health complications; haematuria, kidney damage and cancer in the bladder, at later stages of infection (Colley et al., 2014). Lymphatic filariasis and onchocerciasis helminthic diseases are capable of causing permanent disabilities or even death to host. Furthermore, filariasis and onchocerciasis could cause lymphedema, hydrocoele, elephantiasis, skin disease and blindness (Taylor et al., 2010).

Low birth weight and preterm birth have been linked to Iron deficiency anaemia during gestation as described by Passerini et al. (2012). It was reported that helminthic infections caused by Hookworms can severely contribute to anaemia due to chronic loss of blood (Brooker et al., 2008; Passerini et al., 2012). On the other hand, Larocque and Gyorkos (2006), hypothesized that regular general deworming in developing countries may have a positive impact on birth outcomes if administered during pregnancy, but this is yet to have conclusive evidence. Studies show that helminths infection has a profound effect on the performance of school children and more prevalence among the school age 5–15 years but its prevalence and effect decline in adulthood (Miguel and Kremer, 2004; Larocque and Gyorkos, 2006; Bleakley, 2007). Conclusively, Le Hesran argued that helminthiases might increase host susceptibility to other economically important diseases such as tuberculosis, Malaria and HIV infection (Le Hesran et al., 2004).

## 3 Conclusions

The prevalence of helminthiases is dominant among the poor, children, warm and moist environments. The mode of transmission of helminths is through eggs, larvae, cyst or an intermediate host which aid survival and prevalence of the parasite. The resistance of helminths to drugs is also a survival mechanism.

The remedy for helminths resistance to drugs may derive its source from medicinal plants as these plants consist of mixtures of numerous phytochemical compounds which can initiate multiple biological activities and series of joint actions. Medicinal plants have been tested both *in vitro* and *in vivo* against helminths and there is a prospect for future replacement of the synthetic drugs to medicinal plant in treating worm infections. Infections caused by parasitic worms suffer public neglect in the area of study, medical and research. However, there is currently an enormous benefit for the study of basic and translational helminthology. Anthelminthic interventions have now been recognized as a fundamental human right (Hunt, 2006). Nevertheless, the research in helminthic infections, as well as the cure, still suffer setbacks, compared to other academic niches. This call for both private and public sectors intervention, investment and dedication of more resources for the research and cure of parasitic worms.

## Declarations

### Author contribution statement

All authors listed have significantly contributed to the development and the writing of this article.

### Funding statement

The authors received no funding from an external source.

### Competing interest statement

The authors declare no conflict of interest.

### Additional information

No additional information is available for this paper.
